# U-CARE: Internet-based stepped care with interactive support and cognitive behavioral therapy for reduction of anxiety and depressive symptoms in cancer - a clinical trial protocol

**DOI:** 10.1186/1471-2407-13-414

**Published:** 2013-09-11

**Authors:** Susanne Mattsson, Sven Alfonsson, Maria Carlsson, Peter Nygren, Erik Olsson, Birgitta Johansson

**Affiliations:** 1 Department of Public Health and Caring Sciences, Uppsala University, Box 564, SE-751 22 Uppsala, Sweden; 2 Department of Oncology, Radiology and Radiation Science, Uppsala University, Uppsala, Sweden

**Keywords:** Cancer, Anxiety, Depression, Internet, Psychosocial support

## Abstract

**Background:**

Approximately 20–30% of patients with cancer experience a clinically relevant level of emotional distress in response to disease and treatment. This in itself is alarming but it is even more problematic because it is often difficult for physicians and nurses to identify cancer patients who experience clinically relevant levels of anxiety and depression symptoms. This can result in persistent distress and can cause human suffering as well as costs for individuals and to the community.

**Methods:**

Applying a multi-disciplinary and design-oriented approach aimed at attaining new evidence-based knowledge in basic and applied psychosocial oncology, this protocol will evaluate an intervention to be implemented in clinical practice to reduce cancer patient anxiety and depression. A prospective randomized design will be used.

The overarching goal of the intervention is to promote psychosocial health among patients suffering from cancer by means of self-help programmes delivered via an Internet platform. Another goal is to reduce costs for individuals and society, caused by emotional distress in response to cancer.

Following screening to detect levels of patient distress, patients will be randomized to standard care or a stepped care intervention. For patients randomized to the intervention, step 1 will consist of self-help material, a chat forum where participants will be able to communicate with each other, and a Frequently Asked Questions (FAQ) section where they can ask questions and get answers from an expert. Patients in the intervention group who still report symptoms of anxiety or depression after access to step 1 will be offered step 2, which will consist of cognitive behavioral therapy (CBT) administered by a personal therapist. The primary end point of the study is patients’ levels of anxiety and depression, evaluated longitudinally during and after the intervention.

**Discussion:**

There is a lack of controlled studies of the psychological and behavioral processes involved in this type of intervention for anxiety and depressive disorders. Since anxiety and depressive symptoms are relatively common in patients with cancer and the availability of adequate support efforts is limited, there is a need to develop evidence-based stepped care for patients with cancer, to be delivered via the Internet.

**Trial registration:**

ClinicalTrials.gov Identifier: NCT01630681

## Background

### The need to identify patients with clinical levels of distress

In Sweden approximately 55,000 persons were diagnosed with cancer in 2010 [1]. Research has shown that a large proportion of patients with cancer have the ability to handle the mental strain that the disease can cause [2-4]. However, 20–30% develop anxiety and depression symptoms [5-7]. There is a need for enhanced psychosocial support for this group in order to prevent persistent psychological distress [8]. Patients with incurable disease report higher levels of anxiety and depression compared with patients who can be cured [9]. Several studies have also shown a correlation between cancer patients’ and their family’s levels of psychological distress [10].

Also, a lack of social network and a low socio-economic status, with a low educational level, low income and unfavourable working conditions, can have a negative impact on cancer patients’ psychological and physical wellbeing as well as on disease progression [11,12]. Such factors should be included as potential moderators in studies of interventions aimed at reducing psychological problems.

### Screening to identify patients needing support

There is a need to identify cancer patients who might need extra psychosocial support. It can be difficult to identify psychological problems as they often manifest in the same way as disease-related symptoms, e.g. with fatigue and impaired functioning. Several screening questionnaires are available to identify patients at risk of developing psychological disorders [13]. General screening of patients may identify patients who have an increased need for psychosocial support and psychological treatment [14]. Internet-based screening may be one way to increase access and automate the calculations that are often necessary when screening for psychiatric disorders. Development of interactive computerized systems and Internet-based research into screening with established instruments is an important part of the methodological development.

### Interactive support and cognitive behavioral therapy

During the last decades, computerized interactive support (IS) efforts that combine health information with support for behaviour change, social support such as discussion forums, and support to make decisions have been developed for patients with chronic diseases. A Cochrane review [15] based on 24 randomized controlled trials in chronic disease concluded that such efforts lead to increased knowledge, positive changes in behaviour, a sense of increased social support, better health, improved functional status and fewer symptoms. The conclusions are that this type of support seems efficient, but more studies are needed to confirm that these results also apply to cancer.

Studies that investigate the effects of web-based discussion forums in which patients share information and discuss experiences show conflicting results. One randomized study concluded that an online discussion forum moderated by health professionals can lead to decreased psychological problems in women with breast cancer [16]. These findings were contradicted by the results of a recently published randomized trial in mixed diagnoses that did not show any positive effects of a non-moderated web-based discussion forum, but rather, a tendency towards poorer psychological wellbeing in the intervention group compared with the control group [17].

Using the Internet to deliver cognitive behavioral therapy (CBT) has many advantages. The Internet has become more integrated into the daily lives of a big part of the population and offers new opportunities for interventions. During the past decade, using Internet-delivered interventions has become more common and, according to a meta-analysis including twelve randomized controlled trials, Internet-based CBT has advantages over traditional CBT for both clients and health care providers’ [18]. The treatment can be obtained at any time and place and participants can use it at their own pace and review the material as often as desired. The level of therapist involvement can be adjusted to the actual need and it may be possible to reduce the therapist time while maintaining efficacy. It may also be possible to reach people through the Internet who might otherwise not receive treatment for their problems. According to the same meta-analysis, studies show that Internet-delivered CBT is as effective as traditional, individual, face-to-face CBT [18]. However, there is need for more, and larger, studies to determine the effects of Internet-based CBT for different types of symptoms in patients with cancer.

There is also a need to understand more about the actual uptake of support. A large proportion of cancer patients with psychological problems do not use available supportive psychosocial resources [19]. According to one study, approximately 30% of patients with cancer who reported symptoms of anxiety or depression declined support [20]. Among the motives reported for refraining from making use of the support offered were a long distance from the clinic and that they already had an established support contact. For patients living a long distance from the clinic, Internet-delivered interventions can be a suitable solution.

### Stepped care, a promising route to more individual uptake of support

Stepped care means that care is given with different intensity for different individuals. Treatment effects are repeatedly evaluated and patients who do not respond to one level of support are transferred to another level and receive more intensive support [21]. Stepped care has successfully been used for treatment of anxiety and depression in the elderly and patients with cancer [22,23]. The initial level of stepped care for psychological problems may comprise education about common symptoms and effective self-help strategies (psycho-education), counselling, and support from other patients. A more intensive treatment level may comprise individual CBT. Stepped care can improve access to support and psychological treatment in a cost-effective manner, particularly if the method is provided via the Internet [18,21,24]. There are virtually no studies of the health economic aspects of interventions aimed at reducing psychological distress in patients with cancer. A few studies suggest that treatment with CBT for anxiety, depression and dysfunctional fear of recurrence of cancer is a cost-effective alternative to other methods of treatment [25,26]. It is important to examine the health economic aspects of this type of intervention to determine whether it is cost-effective to implement in routine care.

### Aim

The aim of the planned study is to investigate the effects of an Internet-based stepped care model, comprising IS and CBT, on levels of anxiety, depression and health-related quality of life in adults with cancer and with symptoms of depression and/or anxiety. Further, the aim is to evaluate the health economic aspects of the intervention. Stepped care will be compared with standard care in a randomized study design.

### The main research questions

#### *Primary research question*

• Do participants who receive Internet-based stepped care report lower levels of anxiety and/or depressive symptoms at 10 months after randomization compared with participants receiving standard care? See details under “Statistical power” below.

#### *Secondary research questions*

• Will participants who report anxiety and/or depression symptoms at diagnosis and who receive Internet-based stepped care report lower levels of anxiety, depression, post- traumatic stress and improved health-related quality of life in the short and long term (≤24 months after diagnosis)?

• Is Internet-based stepped care cost-effective or at least cost-neutral compared with standard care at 10 and 24 months after diagnosis?

• To what extent do participants use the IS skills and strategies suggested for countering symptoms?

## Methods

The present project is part of the Uppsala University psychosocial care programme (U-CARE), an interdisciplinary programme which is being conducted in close collaboration between researchers in clinical psychology, information systems, nursing sciences, medical sciences and economics. An Internet platform (the U-CARE portal) has been developed within the U-CARE programme. The U-CARE portal is used for interventions and data collection in all projects undertaken within the framework of the research programme.

### Design

**Figure 1:**
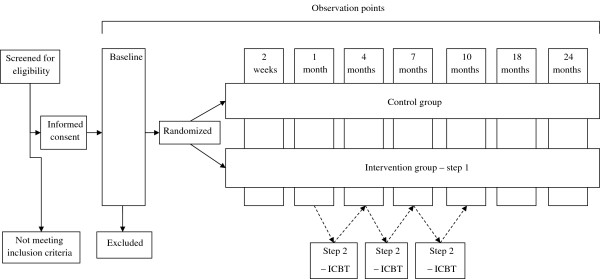
**Flowchart of the study.** ICBT = Internet-based cognitive behavioral therapy.

The present project comprises a prospective randomized controlled trial where stepped care will be compared with standard care. Figure 1 provides an outline of the trial design.

### Participants

Inclusion of patients will start in March 2013 and will continue for approximately 18 months. Patients with newly (within 6 months) diagnosed early-stage breast, prostate or colorectal cancer as well as patients with recurrence of colorectal cancer (within 6 months of diagnosis) at three hospitals in Sweden will be asked to participate in the study. Exclusion criteria are inability to read and understand Swedish, cognitive disability (such as dementia or psychosis), a constant need of care (Karnofsky score <40), short expected survival (<3 months), severe depression or suicide risk with regard to answers on the Montgomery-Åsberg Depression Rating Scale – Self-Report (MADRS-S) measure (see “Data collection” below), and participation in a competing clinical trial including prostate cancer patients receiving radiotherapy. In our previous randomized controlled intervention study on quality of life aspects, approximately 70% of eligible patients agreed to participate, and about 70% of these completed participation [2]. There is a risk that a greater proportion of patients will refuse to take part in the present project since it requires access to the Internet, a mobile phone and some computer skills.

### Statistical power

The main outcome measure is the Hospital Anxiety and Depression Scale (HADS) [27]. It consists of 14 questions divided into one subscale for anxiety symptoms (seven questions) and one for depressive symptoms (seven questions). Both are 21-point scales. Values <7 points on any of the subscales indicate mild anxiety or depression. Values < 10 points indicate clinically significant levels of anxiety and depressive symptoms. A change of at least 20% on the HADS is considered to be a clinically relevant change [28]. In one previous study of patients with <7 points cut-off on any of the HADS subscales, the mean score for anxiety was 10 and for depression, 8 [6]. To achieve 80% statistical power (alpha = 0.05) needed to detect a mean difference of 2.0 points on the anxiety subscale and 1.6 points on the depression subscale, 65 patients are required in each of the intervention and control groups. Based on experience from previous intervention studies, we estimate that approximately 30% of study participants will not complete the data collection. This means that we need to include at least 95 patients in each group. For the present study, approximately 1,300 patients need to be enrolled, <300 of whom are likely to have anxiety or depression symptoms, <200 of whom can be expected to complete the study.

Patients with <7 points on any of the subscales will be allocated to a reference group and answer questionnaires at baseline and at three observation points during the time of the intervention. They will answer questionnaires that measure anxiety, depression and health-related quality of life.

### Randomization

Participants with anxiety and/or depressive symptoms (<7 on any of the HADS subscales) will be randomized to either Internet-based stepped care or standard care. Randomization will be stratified for curable/non-curable disease and will be done in blocks.

### Intervention – stepped care

The intervention involves providing stepped care treatment, which consists of IS (step 1) followed by CBT (step 2). All participants randomized to the intervention group will have access to step 1 from randomization and throughout the study period (24 months). Patients with persistent symptoms of anxiety or depression (<7 on any of the HADS subscales) at 1, 4 or 7 months after inclusion will be offered step 2 and will be able to choose CBT or to have continued access to IS at step 1 only.

### Interactive support – step 1

Step 1 provides information about the disease and about common symptoms and strategies to improve health and prevent physical and psychological problems such as anxiety, sleeping problems, adverse effects of cancer therapy, cancer-related fatigue and pain. Examples of strategies provided include relaxation techniques, physical exercise, good diet and sleeping habits and positive activities. Step 1 consists of web-based material including psycho-education and teaching of simple intervention strategies. It also includes a Frequently Asked Questions (FAQ) database where participants can read questions from other participants as well as ask their own questions. A moderated discussion forum is provided for discussion of various symptoms and how they may be countered, as well as other issues related to the disease or treatment. It is also possible to keep a personal diary and/or write a blog. Doctoral students who are trained nurses have the primary responsibility to answer questions and moderate the discussion forums under the supervision of a psychologist. The moderators may also contact oncologists involved in the study, specialist nurses, a social worker and a dietician if there is need for specialist knowledge or input. Participants who do not log in will be reminded to do so by short messaging service (SMS) or e-mail and offered help with logging in to the portal. The material is collected in a digital library and includes audio and video lectures, downloadable documents, links to relevant websites and suggested reading. The content of the library is divided into modules divided into topics, such as disease treatment, health promotion, and common symptoms with strategies for alleviation.

Representatives from patient organizations have contributed to the design of step 1 of the intervention and will participate in the discussion forum. This is because there must be a relevant content in the discussion forum when the first participants in the randomized controlled trial begin using step 1. Representatives from patient organizations have also participated in tests regarding the usability of the portal.

### Cognitive behavior therapy – step 2

The Internet-based CBT in step 2 consists of standard CBT programmes for psychological problems. At the beginning of treatment in step 2, participants will receive a list of treatment modules regarding sleeping problems, depression, etc and will be able to choose the problem area/s they want to work with. The treatment is highly structured. It follows established treatment manuals and includes exercises, assignments, self-monitoring and weekly contact with a psychologist over the Internet. A participant who does not follow the treatment plan is reminded and encouraged to participate in the programme; however, it is not mandatory to complete the treatment. In total, step 2 consists of 10 weeks of intervention followed by evaluation. Participants cannot repeat step 2. After finishing step 2, participants have, however, access to the material for the remainder of the study period.

### Standard care

Standard care includes the basic information always provided in routine health care, i.e. information about the disease, the treatment that the patient will undergo, possible side effects of the treatment, and what the patient can do to prevent and relieve symptoms and side effects. Information on existing psychosocial support is given to all patients by the physician or nurse in charge. Psychosocial support activities include, on an as-needed basis, the opportunity to talk to a social counsellor or deacon/priest from the hospital church and, occasionally, to support groups. If the need for help with anxiety or depression is identified, patients are encouraged to raise the problems with the physician responsible for the treatment, for further action which might include pharmacological treatment of anxiety and depression, general support and referral to mental health care.

### Data collection

All patient-reported data will be collected through the U-CARE platform. Well-known questionnaires with good psychometric properties will be used. Table 1 presents an outline of questionnaires and observation points.

**Table 1:** Summary of the study’s instruments and the observation points at which they will be used

**Instrument**	**Observation points**
HADS	At baseline and at 1, 4, 7, 10, 18 and 24 months
MADRS-S	At baseline and at 10 months
STAI-S	At baseline and at 10 months
ESSI	At baseline
NLEs	At baseline
EROS	At baseline and at 10 months
PCL-C	At baseline and at 10, 18 and 24 months
EORTC QLQ-C30	At baseline and at 1, 4, 7, 10, 18 and 24 months
EORTC QLQ-BR23^1^	At baseline and at 1, 4, 7, 10, 18 and 24 months
EORTC QLQ-PR25^1^	At baseline and at 1, 4, 7, 10, 18 and 24 months
EORTC QLQ-CR29^1^	At baseline and at 1, 4, 7, 10, 18 and 24 months
FACIT-F	At baseline and at 1, 4, 7, 10, 18 and 24 months
ISI	At baseline and at 1, 4, 7, 10, 18 and 24 months
PTGI-SF	At 10 months
Health economic questionnaire	At 1, 4, 7, 10, 18 and 24 months
EQ-5D	At 2 weeks and 1, 4, 7, 10, 18 and 24 months
Patient satisfaction questionnaire	At 1, 4 and 10 months
Internet use questionnaire	At 1, 4, 7, 10, 18 and 24 months
Knowledge and strategies questionnaire	At 2 weeks and 1 and 10 months

*EORTC QLQ* European Organization for Research and Treatment of Cancer (EORTC) Quality of Life Questionnaire, *EQ-5D* Euroqol 5D, *EROS* Environmental Reward Observation Scale, *ESSI* Enriched Social Support Inventory, *FACIT-F* Functional Assessment of Cancer Therapy: Fatigue, *HADS* Hospital Anxiety and Depression Scale, *ISI* Insomnia Severity Index, *MADRS-S* Montgomery-Åsberg Depression Rating Scale – Self-Report, *NLEs* Negative Life Events questionnaire, *PCL-C* Post-Traumatic Stress Disorder (PTSD) Checklist – civilian version, *PTGI-SF* Post-Traumatic Growth Inventory, Short Form, *QLQ-Br25* Quality of Life Questionnaire with diagnosis-specific modules for breast cancer, *QLQ-C30* Quality of Life Questionnaire, Core 30, *QLQ-CR29* Quality of Life Questionnaire with diagnosis-specific modules for colorectal cancer, *QLQ-PR25* Quality of Life Questionnaire with diagnosis-specific modules for prostate cancer, *STAI-S* State-Trait Anxiety Inventory – State anxiety subscale.

^1^ = if appropriate.

### Anxiety and depression

The HADS [27] is used as a short screening instrument for symptoms of anxiety and depression and for evaluating the effects of the Internet-based stepped care. The HADS has been used extensively both clinically and in cancer care research and has good psychometric properties [29]. It has been successfully administered via the Internet in other projects [30]. The MADRS-S [31,32] is used as a screening instrument for severe depression and/or suicide risk. It has shown good ability to identify depressive symptoms [33] and it will be used in this study to measure symptoms of depression at inclusion and 10 months after inclusion. The MADRS-S consists of nine questions with response scores of 0–60 points, where values <30 points are considered to indicate severe depression. The MADRS-S includes an item connected with suicidal ideation, where scoring ≥3 on this item is considered to be an indication of present suicide risk. Participants scoring ≥3 on this item will be contacted by one of the psychologists of the project and offered adequate help. The Spielberger State-Trait Anxiety Inventory – State anxiety subscale (STAI-S) [34] will be used to measure anxiety at inclusion and 10 months after inclusion. It consists of 20 questions and has good ability to identify anxiety problems in both younger and older patients [35]. The STAI-S has been administered via the Internet in research.

### Post-traumatic stress and health-related quality of life

Post-traumatic stress disorder (PTSD) is measured using the PTSD Checklist – civilian version (PCL-C) [36]. The PCL-C consists of 17 questions regarding trauma-related anxiety symptoms. The questions correspond to the diagnostic criteria specified for PTSD in the Diagnostic and Statistical Manual of Mental Disorders, 4th edition (DSM-IV) [37].

Health-related quality of life will be measured using the European Organization for Research and Treatment of Cancer (EORTC) Quality of Life Questionnaire, Core 30 (QLQ-C30) [38], and EORTC diagnosis-specific modules for breast cancer (QLQ-BR23) [39], prostate cancer (QLQ-PR25) [40] and colorectal cancer (QLQ-CR29) [41]. The diagnosis-specific modules are used as supplements to the QLQ-C30. They are all widely used in research projects in cancer care. The core questionnaire, QLQ-C30, consists of 30 questions measuring global health status, quality of life, five features (physical, emotional, cognitive, social, and role functioning) and nine symptoms that are common to all cancers, regardless of diagnosis. The diagnosis-specific modules consists of 23 (QLQ-BR23), 25 (QLQ-PR25) and 29 (QLQ-CR29) questions measuring functions and symptoms that are specific to each diagnostic group.

### Health economy

For the health economic evaluation, data will be collected from several registers, primarily the longitudinal integration database for health insurance and labour market studies (LISA) or LISA’s original records, the patient register, the multi-generation register, the cancer register, the patient register and the pharmaceutical registry as well as other records that may be relevant. Data on health-related expenses not covered by the registry data will be collected via a project-specific health economic survey (HES). The EuroQol EQ-5D [42] will be used for calculation of life-adjusted life years.

### Medical and socio-demographic background data

Information about the patients’ medical background, treatment and cancer-related outcome will be obtained from the diagnosis-specific Regional Oncology Centre records and from the medical records. Details about the psychosocial support provided within the standard care group will be collected via the HES where the patients will be asked to report any contact with the social worker, psychiatrist, psychologist, psychotherapist or the hospital church. Demographic data will be obtained via project-specific questions.

### Internet use

Data on the intervention and control groups’ Internet use related to their cancer disease will be collected through project-specific questions.

### Knowledge on and use of strategies to counteract symptoms

Knowledge of the disease, common symptoms and strategies to counter symptoms will be examined in a project-specific survey. The survey will include questions about perceived knowledge and whether and to what extent the patients have used, and are using, the proposed strategies to counter current symptoms. In addition, data on each study participant’s use of educational materials, activity in the “Questions and Answers (Q&A)” section, posts in discussion forums and notes in the diary will be logged in the U-CARE portal.

### Observation points

Data will be collected at baseline and at 1, 4, 7, 10, 18 and 24 months after inclusion. For the health economic evaluation, economic data will be collected at the above time points. Data on knowledge and use of strategies as well as health economic data (EQ-5D) will be collected also 2 weeks after inclusion.

### Procedure

#### *Pilot study*

A pilot study will be carried out using the same procedures and design as in the main study (see below). An evaluation will be made after randomization of 20 participants to the study. The evaluation will be made with respect to inclusion frequency, participants’ activity in steps 1 and 2 and the proportion of patients with persistent anxiety and depressive symptoms 1 month after inclusion. If the evaluation does not give rise to changes in the study design or procedures, the pilot study will seamlessly proceed to the main study.

#### *Main study*

Eligible patients at the oncology, urology and surgery clinic will be identified and asked for informed consent at a regular visit to the clinic within 6 months after initial diagnosis or detection of disease relapse.

First, participants will be asked to answer all the study’s questionnaires via the U-CARE portal. Patients with severe depression or suicide risk (as measured by the MADRS-S, see “Data collection”) at inclusion or at the 10-month measurement will be excluded from the project and contacted via telephone by a project staff psychologist. Likewise, patients will be contacted by a psychologist if there is reason to believe that they have severe depression or are suicidal according to what they write in the forum or diary or what they post on the chat. Participants in the intervention group will have access to IS immediately after randomization. Both the intervention and the control group will receive the psychosocial support as part of the standard care at their respective clinic. Both groups will answer a set of questionnaires at observation points described in Table 1. The reference group will answer a set of questionnaires at some of the observation points. The patients will be notified by e-mail of when it is time to answer questionnaires. If they do not complete the questionnaires they will be reminded by e-mail 7 days after baseline and after 7 and 12 days after any of the observation points.

### Data processing and analysis

The end points of the study will be evaluated according to the intention-to-treat principle. In addition the complier average causal effect will be analysed. Differences between the intervention and the control group regarding anxiety, depression, post-traumatic stress, health-related quality of life and the presence and development of these variables over time will be analysed using analysis of variance (ANOVA) with repeated measurements. Should the variables not satisfy the basic assumptions of normal distribution and equal variance, the Mixed regression models will be used for analysis [43]. How and to what extent the different parts of the Internet-based system for psychosocial support are used will be analysed using the data on the study participants’ activities recorded in the system. Qualitative content analysis [44] will be used to analyse the content of the discussion forums and diaries. We also plan to use computerized text analysis. Appropriate methods to control for mass significance will be used.

For process analyses of CBT, multiple regression analysis will be used. The health economic analyses will relate to the interventions’ cost-effectiveness compared with the cost-effectiveness for standard care, which will be analysed in the way that incremental costs are related to the quality-adjusted life years (QALYs). The costs include direct costs (e.g. medical care) and indirect costs (e.g. production) as well as costs for patients, families and health care and to the community. We will analyse the differences between the groups in the short term, after 1, 4, 7, 10 months, and in the long term, after 18 and 24 months. Health economic effects will be analysed after 10 and 24 months. Background variables, such as medical and socio-demographic factors that could potentially function as moderators or confounders, will be controlled for. Internet use other than the IS will be monitored by a project-specific questionnaire administered to both groups in order to control for this potential confounder.

### Ethical aspects

The project has been approved by the Regional Ethical Review Board in Uppsala (Dnr 2012/003). Written informed consent will be obtained from the participants before inclusion. Extensive measures have been taken, and will continue to be taken, to minimize the risk of infringing activities and to ensure that study participants’ personal data cannot be linked to patient-reported data by unauthorized persons. Study participants may at any time terminate participation without giving any explanation. The literature does not suggest that Internet-based support interventions have any negative effects. Rather, there is good reason to assume that different types of Internet-based support and treatment with CBT may be effective for treatment of psychological problems. The main ethical risk is including participants with a need for other specialized medical care, such as those with severe depression. The baseline measurement is designed to minimize that risk. All study participants will also have an established contact with physicians in routine health care.

## Discussion

Most cancer patients have the ability to handle the mental strain that cancer can cause, but a significant proportion of this group report anxiety and depressive symptoms that need to be attended to. Previous support interventions in a general cancer population have not shown convincing or clinically significant effects [2,45]. A plausible explanation is that only patients with elevated levels of psychological distress are helped by the support. We will therefore conduct screening to identify those with clinically relevant levels of distress in order to target the support to those who need it.

Since it has been found that a significant portion of this group do not take part in psychological support offered, a stepwise approach will be used to individualize the level of support needed. In addition, studies have shown that some patients who report emotional distress decline support because, among other reasons, they live a long distance from the clinic. By offering support via the Internet, we may reach a larger population. Studies have also shown that Internet-based CBT can be as effective as in vivo CBT and based on this knowledge it can also be cost-effective to administer assistance via the Internet since the therapist can treat more patients in less time [46].

Internet-based stepped care and the impact of such care for cancer patients with anxiety and depressive symptoms has not been extensively studied previously. There is a lack of controlled studies of the psychological and behavioral processes involved in this type of intervention for anxiety and depressive disorders. Since anxiety and depressive symptoms are relatively common in patients with cancer and the availability of adequate support efforts is limited, there is a need to develop evidence-based stepped care for patients with cancer, to be delivered via the Internet.

To increase the reliability and opportunities to implement the Internet-based psychosocial support nationwide, the study will be carried out in several counties and clinics across Sweden.

A limitation of the design of this study is possible selection bias due to the fact that Internet-based stepped care may appeal more to younger patients and those with computer skills. The study’s reliability can be questioned for the same reason, especially since the design may appeal more to patients with a higher educational level who may be more used to extensive reading. There will also be no blinding in the study and consequently the control group may become under- or overtreated. Furthermore, it should also be considered that the results of this intervention may not be applicable to other countries owing to cultural differences.

## Competing interests

The authors declare that they have no competing interests

## Authors’ contributions

SM contributed to the study design and the development of the intervention and drafted the manuscript. SA contributed to the study design and the development of the intervention and helped to draft the manuscript. MC helped to draft the manuscript.PN contributed to the study design and helped to draft the manuscript. EO contributed to the development of the intervention and helped to draft the manuscript. BJ is the head of the project and was responsible for the development of the study design and the development of the intervention. She also helped to draft the manuscript. All authors read and approved the final manuscript.

## Authors’ information

Susanne Mattsson, Sven Alfonsson, Erik Olsson and Birgitta Johansson: Psychosocial oncology and supportive care group.

## Pre-publication history

The pre-publication history for this paper can be accessed here:

http://www.biomedcentral.com/1471-2407/13/414/prepub

## Original publication

The manuscript contains original unpublished work and is not being submitted for publication elsewhere at the same time. There are no manuscripts based on this protocol under submission in any journal. The protocol has ethical approval from the Regional Ethical Review Board in Uppsala 20120208 (Dnr 2012/003).

## References

[B1] National board OHAW Cancer incidence in Sweden 2011 Official statistics of Sweden, statistics health and medical care 2011 Stockholm: National board of health and welfare

[B2] Johansson B Brandberg Y Hellbom M Persson C Petersson L Berglund G Glimelius B Health-related quality of life and distress in cancer patients: results from a large randomised study Br J Cancer 2008 99 12 1975 1983 10.1038/sj.bjc.6604789 19018255 PMC2607225

[B3] Fann J Thomas-Rich A Katon W Cowley D Pepping M McGregor B Gralow J Major depression after breast cancer: a review of epidemiology and treatment Gen Hosp Psychiatry 2008 30 2 112 126 10.1016/j.genhosppsych.2007.10.008 18291293

[B4] Strong V Waters R Hibberd C Rush R Cargill A Storey D Walker J Wall L Fallon M Sharpe M Emotional distress in cancer patients: the Edinburgh cancer centre symptom study Br J Cancer 2007 96 6 868 874 10.1038/sj.bjc.6603626 17311020 PMC2360098

[B5] Fröjd C Larsson G Lampic C von Essen L Health related quality of life and psychosocial function among patients with carcinoid tumours. A longitudinal, prospective, and comparative study Health and Quality of Life Outcomes 2007 5 1 18 10.1186/1477-7525-5-18 17428340 PMC1852299

[B6] Arving C Glimelius B Brandberg Y Four weeks of daily assessments of anxiety, depression and activity compared to a point assessment with the hospital anxiety and depression scale Qual Life Res 2008 17 1 95 104 10.1007/s11136-007-9275-4 18026852

[B7] van Scheppingen C Schroevers MJ Smink A van der Linden YM Mul VE Langendijk JA Coyne JC Sanderman R Does screening for distress efficiently uncover meetable unmet needs in cancer patients? Psycho-Oncology 2011 20 6 655 663 10.1002/pon.1939 21381148

[B8] Hodges L Humphris G Macfarlane G A meta-analytic investigation of the relationship between the psychological distress of cancer patients and their carers Social Sci & Med 2005 60 1 1 12 10.1016/j.socscimed.2004.04.018 15482862

[B9] Vodermaier A Linden W MacKenzie R Greig D Marshall C Disease stage predicts post-diagnosis anxiety and depression only in some types of cancer Br J Cancer 2011 105 12 1814 1817 10.1038/bjc.2011.503 22095232 PMC3251893

[B10] Hagedoorn M Sanderman R Bolks HN Tuinstra J Coyne JC Distress in couples coping with cancer: a meta-analysis and critical review of role and gender effects Psychological Bull 2008 134 1 1 10.1037/0033-2909.134.1.1 18193993

[B11] Parker PA Baile WF Moor C Cohen L Psychosocial and demographic predictors of quality of life in a large sample of cancer patients Psycho-Oncology 2002 12 2 183 193 12619150 10.1002/pon.635

[B12] Berglund A Holmberg L Tishelman C Wagenius G Eaker S Lambe M Social inequalities in non-small cell lung cancer management and survival: a population-based study in central Sweden Thorax 2010 65 4 327 333 10.1136/thx.2009.125914 20388758

[B13] Pinquart M Duberstein P Depression and cancer mortality: a meta-analysis Psychol Med 2010 40 11 1797 1810 10.1017/S0033291709992285 20085667 PMC2935927

[B14] Vodermaier A Linden W Siu C Screening for emotional distress in cancer patients: a systematic review of assessment instruments JNCI J Natl Cancer Inst 2009 101 21 1464 10.1093/jnci/djp336 PMC3298956 19826136

[B15] Murray E Burns J See TS Lai R Nazareth I Interactive health communication applications for people with chronic disease Cochrane Database Syst Rev 2005 4 12 23 10.1002/14651858.CD004274.pub4 PMC13184810 16235356

[B16] Winzelberg AJ Classen C Alpers GW Roberts H Koopman C Adams RE Ernst H Dev P Taylor CB Evaluation of an internet support group for women with primary breast cancer Cancer 2003 97 5 1164 1173 10.1002/cncr.11174 12599221

[B17] Høybye M Dalton SO Deltour I Bidstrup P Frederiksen K Johansen C Effect of internet peer-support groups on psychosocial adjustment to cancer: a randomised study Br J Cancer 2010 102 9 1348 1354 10.1038/sj.bjc.6605646 20424614 PMC2865756

[B18] Spek V Cuijpers P Nyklícek I Riper H Keyzer J Pop V Internet-based cognitive behaviour therapy for symptoms of depression and anxiety: a meta-analysis Psychol Med 2007 37 03 319 328 10.1017/S0033291706008944 17112400

[B19] Carlson L Angen M Cullum J Goodey E Koopmans J Lamont L MacRae J Martin M Pelletier G Robinson J High levels of untreated distress and fatigue in cancer patients Br J Cancer 2004 90 12 2297 2304 15162149 10.1038/sj.bjc.6601887 PMC2410292

[B20] Thalén-Lindström A Larsson G Glimelius G Johansson B Anxiety and depression in oncology patients; a longitudinal study of a screening, assessment and psychosocial support intervention Acta Oncologica Jan 2013 2013 52 1 118 127 10.3109/0284186X.2012.707785 22934559

[B21] Bower P Gilbody S Stepped care in psychological therapies: access, effectiveness and efficiency. Narrative literature review Br J Psychiatry 2005 186 11 17 10.1192/bjp.186.1.11 15630118

[B22] Fann J Fan M Unützer J Improving primary care for older adults with cancer and depression J Gen Internal Med 2009 24 417 424 10.1007/s11606-009-0999-4 PMC2763171 19838842

[B23] van’t Veer-Tazelaar PJ van Marwijk HWJ van Oppen P van Hout HPJ van der Horst HE Cuijpers P Smit F Beekman ATF Stepped-care prevention of anxiety and depression in late life: a randomized controlled trial Archives Gen Psychiatry 2009 66 3 297 10.1001/archgenpsychiatry.2008.555 19255379

[B24] Marks I Mataix-Cols D Kenwright M Cameron R Hirsch S Gega L Pragmatic evaluation of computer-aided self-help for anxiety and depression Br J Psychiatry 2003 183 1 57 10.1192/bjp.183.1.57 12835245

[B25] Myhr G Payne K Cost-effectiveness of cognitive-behavioural therapy for mental disorders: implications for public health care funding policy in Canada Canadian J Psychiatry 2006 51 10 662 10.1177/070674370605101006 17052034

[B26] Sabariego C Brach M Herschbach P Berg P Stucki G Cost-effectiveness of cognitive-behavioral group therapy for dysfunctional fear of progression in cancer patients Eur J Health Econ 2011 12 5 489 497 10.1007/s10198-010-0266-y 20689977

[B27] Zigmond A Snaith R The hospital anxiety and depression scale Acta Psychiatr Scand 1983 67 6 361 370 10.1111/j.1600-0447.1983.tb09716.x 6880820

[B28] Puhan MA Frey M Büchi S Schünemann HJ The minimal important difference of the hospital anxiety and depression scale in patients with chronic obstructive pulmonary disease Health and Quality of Life Outcomes 2008 6 1 46 10.1186/1477-7525-6-46 18597689 PMC2459149

[B29] Bjelland I Dahl AA Haug TT Neckelmann D The validity of the hospital anxiety and depression scale-an updated literature review J Psychosomatic Res 2002 52 2 69 78 10.1016/S0022-3999(01)00296-3 11832252

[B30] Andersson G Kaldo-Sandström V Ströma L Internet administration of the hospital anxiety and depression scale in a sample of tinnitus patients J Psychosom Res 2003 55 Issue 3 10.1016/s0022-3999(02)00575-5 12932800

[B31] Svanborg P Åsberg M A new self rating scale for depression and anxiety states based on the comprehensive psychopathological rating scale Acta Psychiatr Scand 1994 89 1 21 28 10.1111/j.1600-0447.1994.tb01480.x 8140903

[B32] Svanborg P Asberg M A comparison between the beck depression inventory (BDI) and the self-rating version of the Montgomery asberg depression rating scale (MADRS) J Affective Disord 2001 64 2–3 203 10.1016/s0165-0327(00)00242-1 11313087

[B33] Montgomery SA Asberg M A new depression scale designed to be sensitive to change Br J Psychiatry 1979 134 4 382 10.1192/bjp.134.4.382 444788

[B34] Spielberger CD Gorsuch RL Lushene RE The state-trait anxiety inventory 1970 Palo Alto, Calif: Consulting Psychologists Press Inc

[B35] Kvaal K Ulstein I Nordhus IH Engedal K The Spielberger state‒trait anxiety inventory (STAI): the state scale in detecting mental disorders in geriatric patients Int J Geriatric Psychiatry 2005 20 7 629 634 10.1002/gps.1330 16021666

[B36] Weathers F Litz B Herman D Huska J Keane T The PTSD checklist: reliability, validity, and diagnostic utility. Paper presented at the Annual Meeting of the International Society for 1993 San Antonio, TX: Traumatic Stress Studies

[B37] American Psychiatric Association Diagnostic and Statistical Manual of Mental Disorders 2000 Fourth Washington, DC: American Psychiatric Association Text Revision

[B38] Aaronson NK Ahmedzai S Bergman B Bullinger M Cull A Duez NJ Filiberti A Flechtner H Fleishman SB Haes JCJM The European organization for research and treatment of cancer QLQ-C30: a quality-of-life instrument for use in international clinical trials in oncology JNCI J Natl Cancer Inst 1993 85 5 365 10.1093/jnci/85.5.365 8433390

[B39] Sprangers M Groenvold M Arraras JI Franklin J te Velde A Muller M Franzini L Williams A De Haes H Hopwood P The European organization for research and treatment of cancer breast cancer-specific quality-of-life questionnaire module: first results from a three-country field study J Clin Oncol 1996 14 10 2756 8874337 10.1200/JCO.1996.14.10.2756

[B40] van Andel G Bottomley A Fosså SD Efficace F Coens C Guerif S Kynaston H Gontero P Thalmann G Akdas A An international field study of the EORTC QLQ-PR25: a questionnaire for assessing the health-related quality of life of patients with prostate cancer Eur J Cancer 2008 44 16 2418 2424 10.1016/j.ejca.2008.07.030 18774706

[B41] Whistance R Conroy T Chie W Costantini A Sezer O Koller M Johnson C Pilkington S Arraras J Ben-Josef E Clinical and psychometric validation of the EORTC QLQ-CR29 questionnaire module to assess health-related quality of life in patients with colorectal cancer Eur J Cancer 2009 45 17 3017 3026 10.1016/j.ejca.2009.08.014 19765978

[B42] The EQG EuroQol-a new facility for the measurement of health-related quality of life Health Policy 1990 16 3 199 208 10109801 10.1016/0168-8510(90)90421-9

[B43] Blackwell E de Leon CFM Miller GE Applying mixed regression models to the analysis of repeated-measures data in psychosomatic medicine Psychosom Med 2006 68 6 870 878 10.1097/01.psy.0000239144.91689.ca 17079709

[B44] Holsti OR Content analysis for the social sciences and humanities 1969

[B45] Arving C Sjoden PO Bergh J Hellbom M Johansson B Glimelius B Brandberg Y Individual psychosocial support for breast cancer patients: a randomized study of nurse versus psychologist interventions and standard care Cancer nursing 2007 30 3 E10 E19 10.1097/01.NCC.0000270709.64790.05 17510577

[B46] Wright JH Wright AS Albano AM Basco MR Goldsmith LJ Raffield T Otto MW Computer-assisted cognitive therapy for depression: maintaining efficacy while reducing therapist time Am J Psychiatry 2005 162 6 1158 1164 10.1176/appi.ajp.162.6.1158 15930065

